# Future Medical Artificial Intelligence Application Requirements and Expectations of Physicians in German University Hospitals: Web-Based Survey

**DOI:** 10.2196/26646

**Published:** 2021-03-05

**Authors:** Oliver Maassen, Sebastian Fritsch, Julia Palm, Saskia Deffge, Julian Kunze, Gernot Marx, Morris Riedel, Andreas Schuppert, Johannes Bickenbach

**Affiliations:** 1 Department of Intensive Care Medicine University Hospital RWTH Aachen Aachen Germany; 2 SMITH Consortium of the German Medical Informatics Initiative Leipzig Germany; 3 Jülich Supercomputing Centre Forschungszentrum Jülich Jülich Germany; 4 Institute of Medical Statistics, Computer and Data Sciences Jena University Hospital Jena Germany; 5 School of Natural Sciences and Engineering University of Iceland Reykjavik Iceland; 6 Institute for Computational Biomedicine II University Hospital RWTH Aachen Aachen Germany

**Keywords:** artificial intelligence, AI, machine learning, algorithms, clinical decision support, physician, requirement, expectation, hospital care

## Abstract

**Background:**

The increasing development of artificial intelligence (AI) systems in medicine driven by researchers and entrepreneurs goes along with enormous expectations for medical care advancement. AI might change the clinical practice of physicians from almost all medical disciplines and in most areas of health care. While expectations for AI in medicine are high, practical implementations of AI for clinical practice are still scarce in Germany. Moreover, physicians’ requirements and expectations of AI in medicine and their opinion on the usage of anonymized patient data for clinical and biomedical research have not been investigated widely in German university hospitals.

**Objective:**

This study aimed to evaluate physicians’ requirements and expectations of AI in medicine and their opinion on the secondary usage of patient data for (bio)medical research (eg, for the development of machine learning algorithms) in university hospitals in Germany.

**Methods:**

A web-based survey was conducted addressing physicians of all medical disciplines in 8 German university hospitals. Answers were given using Likert scales and general demographic responses. Physicians were asked to participate locally via email in the respective hospitals.

**Results:**

The online survey was completed by 303 physicians (female: 121/303, 39.9%; male: 173/303, 57.1%; no response: 9/303, 3.0%) from a wide range of medical disciplines and work experience levels. Most respondents either had a positive (130/303, 42.9%) or a very positive attitude (82/303, 27.1%) towards AI in medicine. There was a significant association between the personal rating of AI in medicine and the self-reported technical affinity level (H_4_=48.3, *P*<.001). A vast majority of physicians expected the future of medicine to be a mix of human and artificial intelligence (273/303, 90.1%) but also requested a scientific evaluation before the routine implementation of AI-based systems (276/303, 91.1%). Physicians were most optimistic that AI applications would identify drug interactions (280/303, 92.4%) to improve patient care substantially but were quite reserved regarding AI-supported diagnosis of psychiatric diseases (62/303, 20.5%). Of the respondents, 82.5% (250/303) agreed that there should be open access to anonymized patient databases for medical and biomedical research.

**Conclusions:**

Physicians in stationary patient care in German university hospitals show a generally positive attitude towards using most AI applications in medicine. Along with this optimism comes several expectations and hopes that AI will assist physicians in clinical decision making. Especially in fields of medicine where huge amounts of data are processed (eg, imaging procedures in radiology and pathology) or data are collected continuously (eg, cardiology and intensive care medicine), physicians’ expectations of AI to substantially improve future patient care are high. In the study, the greatest potential was seen in the application of AI for the identification of drug interactions, assumedly due to the rising complexity of drug administration to polymorbid, polypharmacy patients. However, for the practical usage of AI in health care, regulatory and organizational challenges still have to be mastered.

## Introduction

While a balance between the increasing amount of documented data on the one hand and the demographic change and aging populations on the other hand challenges our health care systems, big data and artificial intelligence (AI) in medicine offer a huge potential to relieve physicians from the increasing complexity of today’s health care and information overload when treating patients [1,2]. Over the last decade, research on AI in medicine and biomedicine and the number of publications in these fields have substantially increased [3,4]. Research has come up with promising AI developments in general machine learning (ML) algorithms, for manifold applications to predict clinical events, to improve diagnoses accuracy as well as treatments, and to reduce the burden of disease [5,6]. Well-known examples for AI in medicine are the application of deep learning as a subfield of ML in medical imaging for disease detection from x-rays [7]. Thus, it is expected that AI in medical practice will meet higher expectations of medical treatment and physicians and will increase the efficiency of clinical care. AI is perceived as the next big thing that will sustainably change medicine towards precision and personalized medicine [8] and change health care and with it, the role of physicians. It is believed that physicians will not be replaced by AI, but AI will make lives easier and focussed where human interaction is really required.

### Big Data and AI in Medicine: Definition and Application Areas

An ever-increasing amount of medical data is being recorded by monitoring patient care devices, enabling big data analysis in health care [9]. This paves the way for the application of different ML techniques like deep learning [7], traditional shallow neural networks, support vector machines, and random forests, which are specific models for using AI in practice [10]. Besides conventional ML techniques, deep learning in particular offers advantages for understanding and manipulating the highly relevant class of data, especially for images, language, and speech recognition [11]. However, while deep learning is superior for specific applications, it has limitations for other applications where conventional ML techniques are superior [12]. Examples include cases where large datasets are not available to study a specific medical condition, as deep learning generally requires a large dataset to perform well in practice.

Despite its widespread use, a holistic definition of the term “artificial intelligence” is challenging. This can be partly explained by the fact that it is a “high-level” term, often not mentioning concrete ML algorithms or models in a clear context. Examples of AI in medicine are AI applications to support diagnostic procedures, predict the course of the disease [13-17], enhance the potential of clinical decision support [18], and support the management of hospital workflows [19,20]. Thereby, AI offers the possibility to support physicians in delivering high-quality medicine and increasing medical care efficiency.

### Preconditions for AI Development for Health Care

One essential precondition for the development of AI in medical practice is data availability to develop and train the algorithms. Therefore, the creation of research databases with consolidated anonymized patient data, ideally from multiple locations making clinical routine data available for so called “secondary usage,” is desirable. One very prominent example of such a database is the freely accessible critical care database MIMIC-III (Medical Information Mart for Intensive Care) [21]. After accepting a data use agreement, researchers are granted unrestricted access for analysis. Since its publication in August 2015, the respective publication was cited more than 850 times, showing the great interest in this database and its wide scientific usage [22]. Nevertheless, due to different health care systems and information systems used for patient care, the structure and content of patient data from German hospitals are not in accordance with data from US hospitals. Consequently, there is an urgent need to establish research databases that are applicable to the situation in Germany.

### Challenges of Big Data and AI in Medicine

Today, the widespread practical implementation of AI and AI-based decision support into hospital care has not yet become reality [23]. Especially in radiology and other medical imaging procedures, vendors of medical technology have predominantly integrated some kind of AI (eg, ML algorithms) into their products [24]. In addition, in the field of medical prediction, there are cases that have already been applied to electronic health records to complete prospective verification studies [25,26]. Cabitza et al [27] described the sociotechnical elements that must be mastered to successfully implement potentially effective AI in real-world clinical settings, which they call the “last mile” gap of AI implementation. Further multifaceted technical, regulatory, social, and human factors hinder the practical application of AI in clinical care [23].

Tremendous efforts have already been made to evaluate AI in health care and AI-enabled clinical decision support [28]. However, only a few medical AI applications are really used in clinical practice. As physicians are supposed to be the primary users of AI in medicine, there is a need to investigate physicians’ requirements and expectations for future developments of AI in health care. In addition, regulatory questions, like the liability for medical errors, must be regulated if AI is to be used in physician practice and patient care [29].

Another often observed challenge in applying AI is the availability of sensitive patient datasets due to General Data Protection Regulation constraints, mostly when AI is used beyond just one health care organization. In this context, federated ML [30] is a promising approach to obtain powerful, accurate, safe, robust, and unbiased models by enabling multiple organizations to train collaboratively without the need to exchange or centralize datasets. Also, a federated ML approach can be useful where datasets within one organization might not be enough to train good models (eg, rare diseases). In this context, transfer learning approaches [31] are feasible, too, whereby medical AI models are created using a pretrained, state-of-the-art AI model from a different larger medical dataset or otherwise openly available data (eg, ImageNet dataset).

Both are not often applied in Germany today. To address the multifaceted complexity of AI, the purpose of our study was to evaluate the general perception towards AI in medicine among physicians, but also towards concrete application and the opinion on the usage of anonymized patient data for (bio)medical research and AI development.

To achieve this, we developed a web-based survey for physicians in German academic hospitals. The results should also help researchers and data scientists to better understand physicians’ needs regarding AI systems and to boost their use in clinical care.

## Methods

### Study Design, Data Collection, and Recruitment

For the survey's conceptualization, open and explorative interviews were carried out with 3 junior and 3 senior physicians. The results were structured and then utilized to frame the survey questions in German. These questions were integrated into an open web-based survey (LimeSurvey) to be conducted among our study population in 8 German university hospitals consisting exclusively of physicians from the full range of medical disciplines. The local ethics committee and local data protection officer did not express objections to the operationalization of the web-based survey. On the first page of the survey, we informed the participants about the length, purpose, and expected time to fill in the questionnaire. As participants were free to participate and contribute to the study, we regarded the survey's completion as consent for the usage, analysis, and publication of the collected survey data. For verification and functionality validation, we performed a test phase of the online survey with 25 anesthesiologists and critical care physicians in June 2019. Minor adaptations were added in the final survey version before its link was sent to physicians in 8 university hospitals via email by local persons in charge. The survey was available for 19 weeks from June 2019 till October 2019. Within the data collection period, no content modifications nor bug fixes were necessary, and we did not identify any unforeseen events like system errors or server downtime.

The survey was separated into 2 sections. The first section was comprised of questions about AI in medicine, and the second contained general biographical questions.

A translated, English version of the survey is attached in Multimedia Appendix 1. We only included completely filled out questionnaires in the statistical analysis. However, this might include some questions that were not answered (no response).

The questions about AI in medicine are separated into 3 sections: (1) personal opinion about AI in health care (Q1.1-Q1.16; Q4), (2) fields of application of AI in medicine (Q2.1-Q2.25), (3) usage of anonymized patient data for research purposes (Q3.1-Q3.4)

The question groups 1, 2, and 3 were phrased as single-choice questions asking physicians about their personal view on given statements using a 4-point Likert scale without a neutral option. The first set of questions (Q1.1–Q1.16) in the survey explored the attitudes towards AI in medicine. The second set of questions focused on fields of AI application in medicine. In total, 25 AI applications were given, and physicians were asked to rate if the proposed applications could substantially improve patient care in the future. The third set of questions explored physicians' opinions on the secondary usage of anonymized patient data for research purposes (eg, for AI development for medical practice). Physicians were asked whether they agreed or disagreed with the statements on the usage of anonymized patient data for clinical and biomedical research. Finally, we asked physicians how positively or negatively they evaluated the use of AI in medicine on a 5-point Likert scale. This question (Q4) was assigned to the first section, “Personal opinion about AI in health care.” Biographical answers were mostly conceptualized as closed-ended, single-choice questions (eg, demographic questions), but were also presented as multiple choice questions (eg, medical discipline and predominant workplace).

### Statistical Analysis

For the statistical analysis, we stratified the potential fields of AI applications in medicine into 6 categories: (1) imaging procedures, (2) other diagnostic procedures, (3) intensive care unit (ICU)/anesthesia, (4) medication and therapy, (5) workflow support and education, (6) prognosis assessment (Textbox 1). Here, we partly reference peer-reviewed publications of AI algorithms, comparing AI algorithms with physicians cited by Topol [32]. In addition, we categorized the 33 medical disciplines into 6 categories for further subgroup analysis (see Table S1 in Multimedia Appendix 2). We analyzed most of the data descriptively using graphics produced by the R packages sjPlot [33] and ggplot2 [34]. Where appropriate in the survey data analysis, we conducted Kruskal-Wallis tests to investigate the relationship between AI rating and biographical data. All analyses were conducted in R version 4.0.3 [35].

**Categories of artificial intelligence (AI) applications in stationary hospital care.**

- 1. Imaging procedures
  1.1 Analysis of x-rays, computed tomography (CT), magnetic resonance tomography (MRT), sonographies [36-38]
  1.2 Analysis of histopathologic fine cuts [39,40]
  1.3 Analysis of endoscopic pictures or videos [41-43]
  1.4 Analysis of dermatologic reflected light microscopy [44,45]

- 2. Other diagnostic procedures
  2.1 Analysis of electroencephalography (EEG)/electrocardiography (ECG) [46,47]
  2.2 Diagnosing rare diseases [48,49]
  2.3 Triage in emergency care [50,51]
  2.4 Diagnosing psychiatric diseases [52,53]
  2.5 Subspecification of hematologic diseases [54-56]

- 3. Intensive care unit (ICU)/anesthesia
  3.1 Early alarm of the deterioration of patient status [57]
  3.2 Reduction of false alarms in intensive care medicine [58]
  3.3 Automatic mechanical ventilation [59,60]
  3.4 Support of parenteral or enteral nutrition [61]
  3.5 Automatic anesthesia administration [62]

- 4. Medication and therapy
  4.1 Oncologic therapy planning [18,63]
  4.2 Antibiotic stewardship [64]
  4.3 Identification of drug interactions [65-67]
  4.4 Medication for geriatric patients [68]
  4.5 Medication for pediatric patients [69]

- 5. Workflow support and education
  5.1 Education and training of medical students and physicians [70]
  5.2 Workflow support in stationary hospital care [19,20]
  5.3 Medical recording or discharge letters [71,72]

- 6. Prognosis assessment
  6.1 Prediction of effects of therapeutic interventions [73]
  6.2 Assessment of prognosis of malignant diseases [13-15]
  6.3 Assessment of prognosis of nonmalignant diseases [16,17]

## Results

### Demographic and Professional Characteristics

The online survey was finished by 121 (121/303, 39.9%) female and 173 (173/303, 57.1%) male physicians (no response: 9/303, 3.0%). Their mean length of clinical work experience was 12.7 years. In particular, physicians from the age groups of 25-34 years (98/303, 32.3%), 35-44 years (103/303, 34.0%), and 45-54 years (69/303, 22.8%) participated. Physicians from a wide range of medical disciplines and from all proposed clinical hierarchical levels took part in the survey (Table 1). Additionally, all proposed operational areas (hospital ward, operating theater, outpatient clinic, ICU, office, laboratory, functional area, others areas) are represented in the survey (Table 1).

**Table 1:** Demographic and professional characteristics.

Characteristic		Values (n=303)
**Age range (years), n (%)**		
	18-24	1 (0.3)
	25-34	98 (32.3)
	35-44	103 (34.0)
	45-54	69 (22.8)
	55-65	21 (6.9)
	>65	3 (1.0)
	No response	8 (2.6)
**Gender, n (%)**		
	Female	121 (39.9)
	Male	173 (57.1)
	No response	9 (3.0)
**Current occupation, n (%)**		
	Assistant physician	101 (33.3)
	Medical specialist	49 (16.2)
	Senior physician	108 (35.6)
	Clinic director	28 (9.2)
	Others	6 (2.0)
	No response	11 (3.6)
**Medical field or discipline, n (%)**		
	Anesthesiology/intensive care medicine	75 (24.8)
	Internal medicine	53 (17.5)
	Pediatrics	25 (8.3)
	Surgery	22 (7.3)
	Neurology	14 (4.6)
	Dermatology	12 (4.0)
	Microbiology, virology, infectiology	10 (3.3)
	Psychiatry and psychotherapy	10 (3.3)
	Psychosomatic medicine and psychotherapy	8 (2.6)
	Neurosurgery	8 (2.6)
	Ophthalmology	7 (2.3)
	Pathology	7 (2.3)
	Otorhinolaryngology	5 (1.7)
	Child and adolescent psychiatry and psychotherapy	5 (1.7)
	Laboratory medicine	5 (1.7)
	Radiology	5 (1.7)
	Urology	5 (1.7)
	Other disciplines/ specialization	43 (14.2)
**Predominant workplace, n (%)**		
	Hospital ward	123 (40.6)
	Operating theater	106 (35.0)
	Outpatient clinic	100 (33.0)
	Intensive care unit	89 (29.4)
	Office	50 (16.5)
	Laboratory	33 (10.9)
	Functional area	30 (9.9)
	Others	15 (5.0)
Clinical professional experience (years), mean (SD)		12.7 (9.3)

### Physicians’ Attitudes Towards AI in Medicine

A majority of physicians reported either a positive (130/303, 42.9%) or a very positive attitude (82/303, 27.1%) towards AI in medicine (Q4; see Multimedia Appendix 3), representing more than two-thirds of the respondents; 18.2% (55/303) rated it neutral, and just 5.6% (17/303) rated it either negative or very negative.

**Figure 1:**
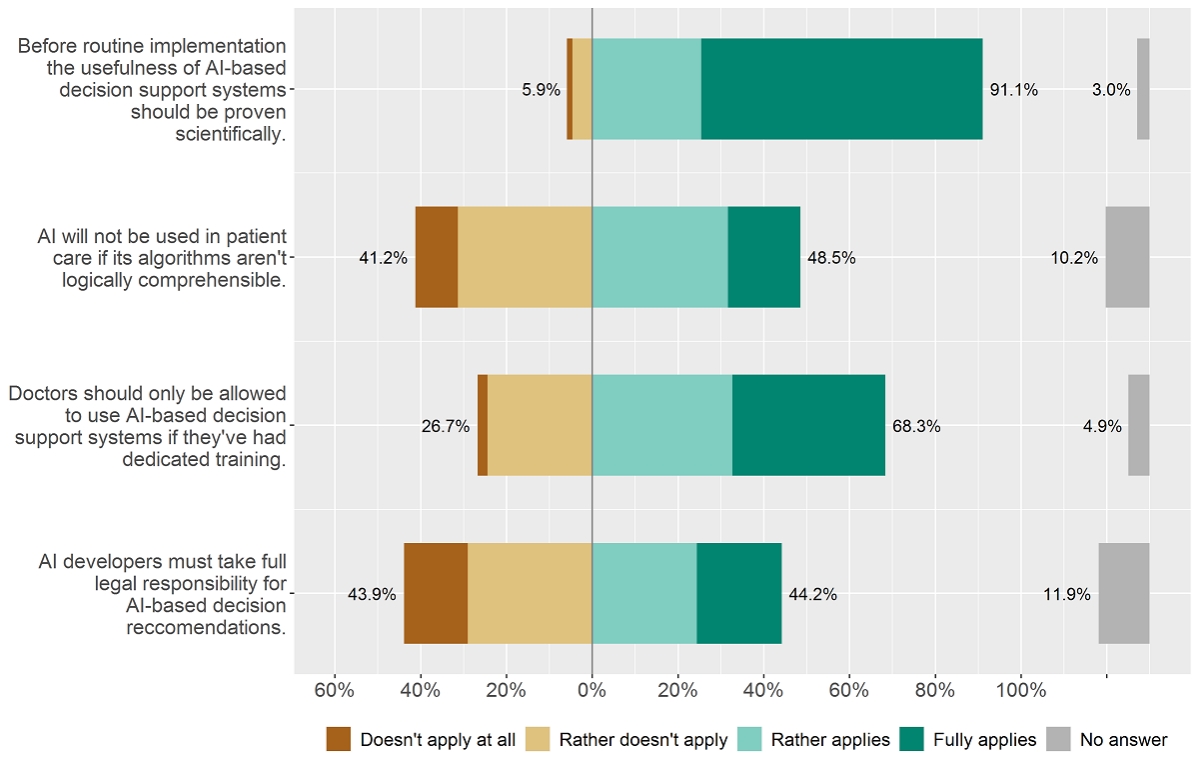
Rules and regulatory requirements of artificial intelligence (AI) usage in medicine.

As described in the Methods, we categorized the first question group into 3 subcategories. The first category focused on the rules and regulatory requirements of AI in medicine (see Figure 1). We found strong agreement (ie, “rather applies” and “fully applies”) among physicians (276/303, 91.1%) for a scientific evaluation before the implementation of an AI-based system. Furthermore, the requirement for special “AI training” for physicians before usage of an AI-based decision support system was clearly favored (207/303, 68.3%).

The statements regarding the responsibility for AI decisions and the influence of algorithms' lack of logical comprehensibility returned a much less clear picture with answers split nearly in half.

**Figure 2:**
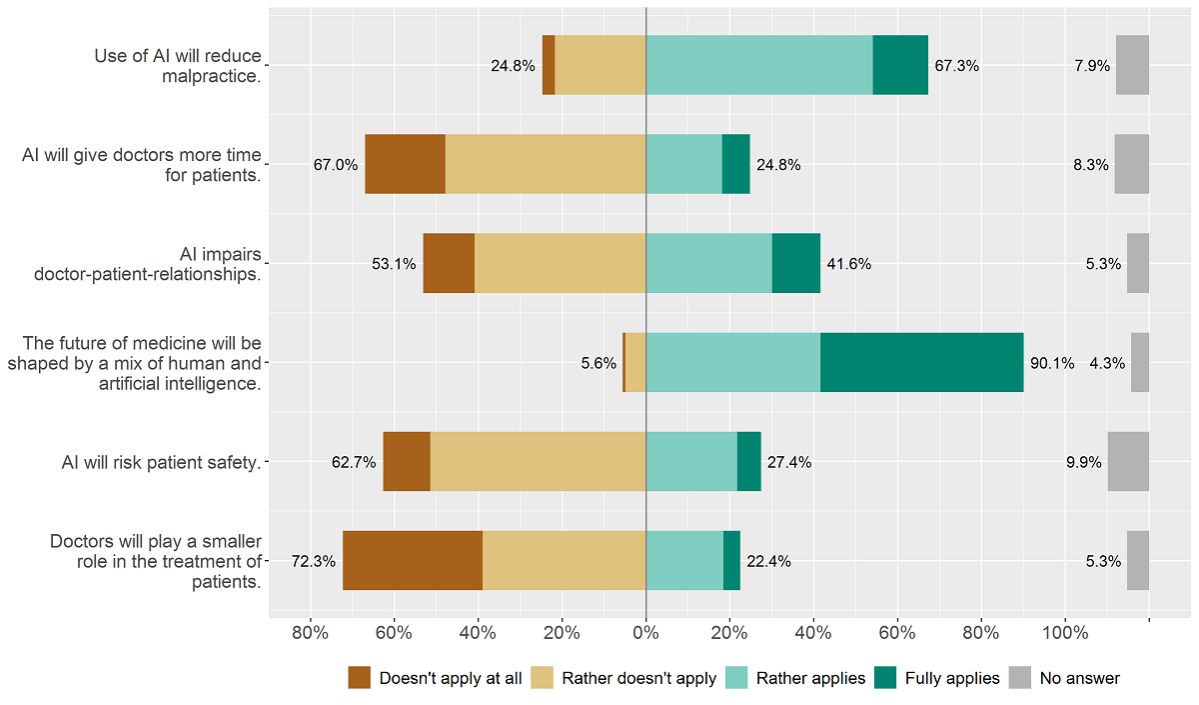
Effect of artificial intelligence (AI) on medical treatment.

In the second subcategory of questions, “effect of AI on medical treatment” (see Figure 2), respondents mostly agreed with the statement “The future or medicine will be shaped by a mix of human and artificial intelligence” (273/303, 90.1%). Most physicians also expected a reduction of malpractice through the use of AI (204/303, 67.3%). At the same time, a majority didn’t expect (ie, “doesn’t apply at all” and “rather doesn’t apply”) that AI would give them more time for their patients (203/303, 67.0%) or that they would play a minor role in the treatment of patients (219/303, 72.3%).

**Figure 3:**
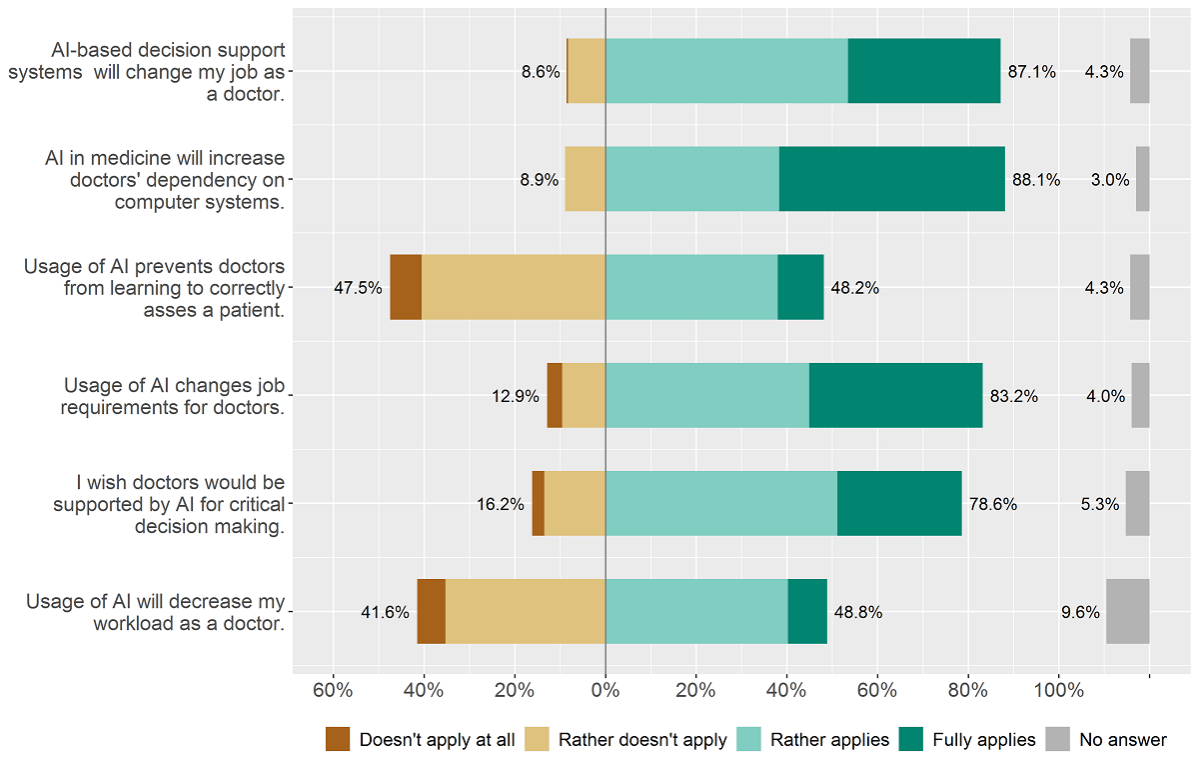
Effect of artificial intelligence (AI) on physicians’ work.

The third subcategory of questions (see Figure 3) focussed on the effect of AI in physicians’ work. Here, respondents agreed that the usage of AI in health care would increase physicians’ dependence on computer systems (267/303, 88.1%). They also agreed that AI-based decision support systems would change their work as a physician (264/303, 87.1%). The clear majority also anticipated a change of physicians’ job requirements (252/303, 83.2%). For the statement “Usage of AI prevents doctors from learning to correctly assess a patient,” agreement and disagreement were roughly evenly distributed (agreement: 146/303, 48.2%; disagreement: 144/303, 47.5%).

The Kruskal-Wallis test revealed no significant difference in the personal rating of AI between the current occupations of assistant physician, medical specialist, senior physician, and clinic director (H_3_=6.39, *P*=.09; see Multimedia Appendix 4). We could also not find a strong association between the AI score and the medical discipline groups described in the Methods section (H_5_=5.92, *P*=.31; see Multimedia Appendix 5).

**Figure 4:**
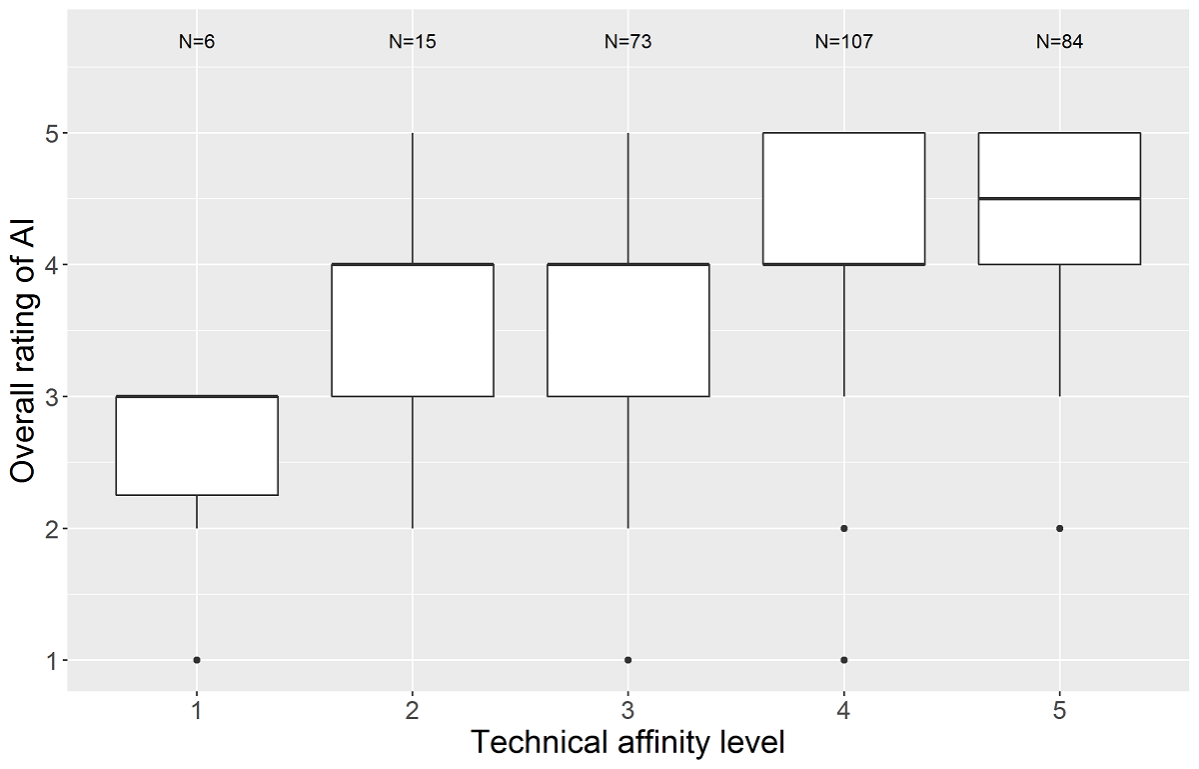
Personal rating of artificial intelligence (AI) stratified by technical affinity score.

As expected, we found a significant association between the personal rating of AI in medicine and the self-reported technical affinity level (H_4_=48.3, *P*<.001; Figure 4).

### AI in Medicine: Fields of Application With the Potential to Improve Clinical Practice

**Figure 5:**
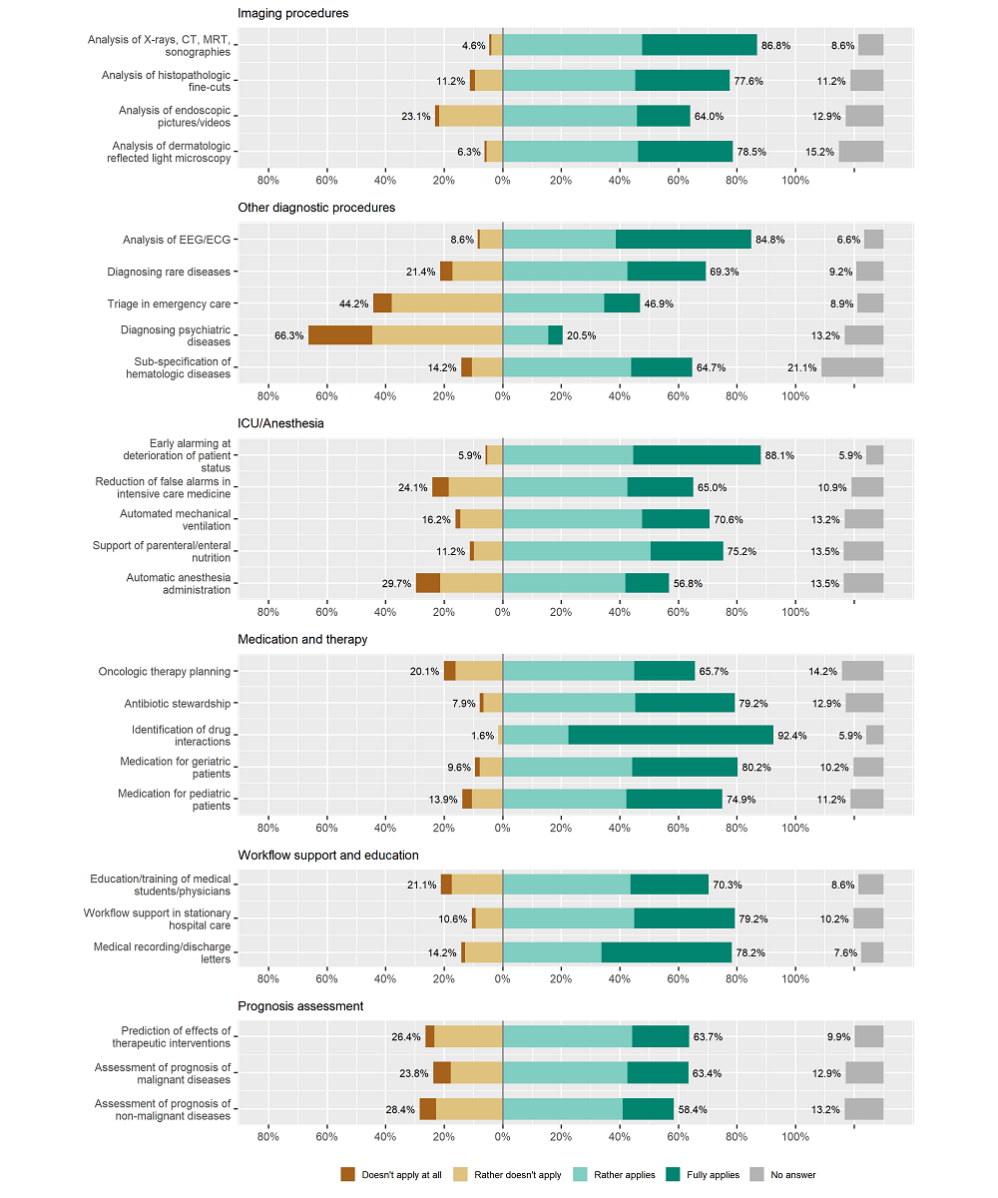
Artificial intelligence (AI) applications and potential for the future of medicine. CT: computed tomography; ECG: electrocardiogram; EEG: electroencephalogram; MRT: magnetic resonance tomography.

In the second section of the survey, we asked the physicians for their appraisal of the potential of AI in medicine to improve clinical care in various fields of application. In total, 25 AI applications were proposed. As described in the Methods section, we also stratified the applications for analysis into 6 categories (Textbox 1, Figure 5).

In the first category, “AI for imaging procedures,” a large majority of physicians agreed that all proposed applications had the potential to improve patient care substantially in the future. There was especially high agreement among respondents for the potential of AI to enhance the analysis of x-rays, computed tomography, magnetic resonance tomography, and sonographies (263/303, 86.8%). However, there was less agreement for the future potential of AI in the analysis of endoscopic images and videos (194/303, 64.0%) than for the other applications.

In the second category, “AI for other diagnostic procedures,” most physicians expected patient care to be improved significantly by using AI for the analysis of electroencephalograms and electrocardiograms and subspecification of hematologic diseases (257/303, 84.8%). Only a minority of respondents saw a role of AI in the diagnosis of psychiatric diseases (62/303, 20.5%) and in triage in emergency care (142/303, 46.9%).

The application of AI for ICU and anesthesia was assessed in the third category of AI applications for medicine. The majority of physicians agreed that all applications would improve patient care, even though the agreement for the potential of automatic anesthesia administration (172/303, 56.8%) was rated much lower than for the application of AI for an early alarm of the deterioration of patient status (267/303, 88.1%).

Furthermore, in the fourth and fifth categories “AI for medication and therapy” and “AI for workflow support and education,” respectively, a majority of respondents expected an improvement of daily practice through the listed AI applications. While AI's potential for the identification of drug interactions was outstandingly high (280/303, 92.4%), fewer physicians were convinced that AI for oncology therapy planning will advance patient care (199/303, 65.7%). Also, workflow support in stationary hospital care was expected and rated as beneficial for patient care in the future (240/303, 79.2%).

In the sixth category, regarding usage of AI for prognosis assessment, therapeutic interventions, and prognosis of malignant and nonmalignant diseases, we received lower agreement than in most other categories regarding an expected improvement in patient care.

In sum, in almost all categories of AI applications in medicine, physicians saw a high potential to improve patient care. A ranking of all proposed applications across all categories of applications with the highest number of answers for ”rather applies” and “fully applies” can be found in Table S2 in Multimedia Appendix 2. A short version, including the highest-rated applications and the lowest-rated potential to improve patient care in the future, is presented in Table 2. This table shows that the most frequently mentioned application to improve patient care was “Identification of drug interactions.” On the other side of the scale, the 2 least often mentioned AI applications with potential for the future of health care were the usage of AI for “diagnosis of psychiatric diseases” and for “triage in emergency care.” Beside those applications, there were also high numbers of “no responses” for specific applications like the application of AI for “Subspecification of hematologic diseases” (No response: 64/303, 21.1%).

**Table 2:** Applications with the highest-rated and lowest-rated potential to improve patient care in the future.

Rating	Artificial intelligence (AI) application	Field of application	Responses of “rather applies“ or “fully applies“, n (%)
1	Identification of drug interactions	Medication and therapy	280 (92.4)
2	Early alarming of deterioration of patient status	ICU^a^/anesthesia	267 (88.1)
3	Analysis of x-rays, CT^b^, MRT^c^, sonographies	Imaging procedures	263 (86.8)
4	Analysis of ECGs^d^ and EEGs^e^	Other diagnostic procedures	257 (84.8)
[...]			
22	Assessment of prognosis of nonmalignant diseases	Prognosis assessment	177 (58.4)
23	Automatic anesthesia administration	ICU/anesthesia	172 (56.8)
24	Triage in emergency care	Other diagnostic procedures	142 (46.9)
25	Diagnosis of psychiatric diseases	Other diagnostic procedures	62 (20.5)

^a^ICU: intensive care unit.

^b^CT: computed tomography.

^c^MRT: magnetic resonance tomography.

^d^ECG: electrocardiogram.

^e^EEG: electroencephalogram.

### Personal Opinion of Physicians on the Secondary Usage of Anonymized Patient Data for Medical Research

**Figure 6:**
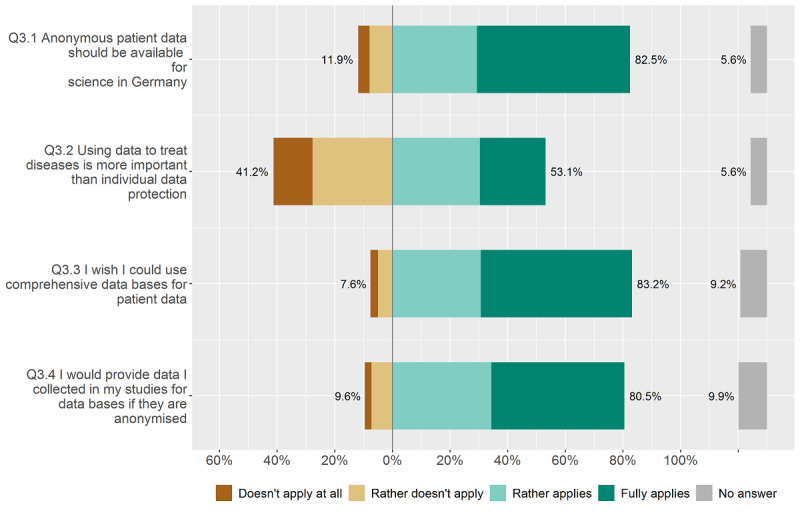
Attitude on the usage of (anonymized) patient data for medical research.

In the third group of questions, we investigated physicians' attitudes on the secondary usage of anonymized patient data for medical data research, which in sum, was positive for the majority of respondents (see Figure 6). Solely for the question regarding balancing better treatment of diseases versus individual data protection, controversial answers were given. Most physicians thought that anonymized patient data should be freely available in Germany for research purposes (250/303, 82.5%), and 83.2% of respondents (252/303) agreed or fully agreed that they wished to be able to use anonymized patient data for their own research.

## Discussion

### Principal Findings

In our study, we could demonstrate that most physicians reported a positive or a very positive attitude towards AI in medicine. Physicians expect that AI will be used in clinical practice for various applications and will substantially improve patient care. There was agreement among the majority of physicians that AI will change their work as a physician. Participants also had a positive attitude towards using anonymized patient data for research purposes as a precondition for the development of algorithms for medical practice. Nevertheless, the usage of AI in medicine in today’s clinical practice in hospitals and health care in Germany is rare.

Shaw et al [74] investigated the challenges of implementing AI in health care compared with other technologies, suggesting that AI implementation in medicine poses novel difficulties. Specific hurdles to be mastered are the absence of interoperabilitiy standards and missing regularities in cases of AI-driven, wrong decisions [23].

To overcome the problem of missing interoperability and improve access to health care data for clinical and biomedical research in Germany, the Ministry of Research and Education has initiated the German Medical Informatics Initiative to make clinical health data from patient care available for medical research [75]. Four consortia (Smart Medical Information Technology for Healthcare [SMITH] [76], Medical Informatics in Research and Care in University Medicine [MIRACUM] [77], HiGHmed [78], and Data Integration for Future Medicine [DIFUTURE] [79]) that include all German university hospitals are conceptualizing, developing, and operating so-called data integration centers in the university medical centers to make health care data from health information systems accessible for medical research. Beyond that, the initiative aims to create the regulatory framework and prerequisites for the secondary usage of routine health care data for (bio)medical data research [75].

### Physicians’ Attitudes Towards AI in Medicine

Physicians participating in our survey had a positive attitude towards the usage of AI in medicine. Nevertheless, they emphasized the need for scientific proof prior to broad implementation of AI-based systems. Besides the obligatory medical device instruction for AI in medicine, physicians want to have dedicated professional training to use AI in medicine. Another precondition for clinical usage of AI is clarity about the legal liability for its usage, especially when the basis of AI recommendations might not be easy to comprehend at once. Our survey's controversial answers to legal and regulatory questions show that rules and regulatory requirements are either not clear or nonexistent. Sullivan and Schweikart [29] described the complexity of legal responsibilities of health professionals and technology manufacturers, especially if the AI technology recommendations are not explainable. The authors highlighted one major problem: If the reasoning for recommendations is unknown, the AI is a black box, and there is a need for new legal solutions for AI usage in medicine.

While most physicians expect the future of medicine to be characterized by the combination of human and artificial intelligence, AI has already been proven to be able to outperform human physicians in specific tasks [80]. Nevertheless, human intelligence also learns from AI systems. As a restriction, the authors think that an AI system making fully autonomous decisions would be neither desirable nor acceptable for the public.

Therefore, a hybrid solution of human and artificial intelligence can form a symbiotic relationship. Physicians expect that AI will significantly impact them and introduce changes for their daily work. This also includes the dependency of physicians on computer systems and new job requirements for physicians. Yu et al [81] described that AI can improve the quality of care by reducing human error and reducing human fatigue from a routine clinical task, but will probably not reduce physicians' workload, because medical guidelines might suggest higher frequencies of examinations for vulnerable patients. The authors Magrabi et al [28] described the challenges of evaluating AI-based decision support and AI's practical implications for medical practice. Due to the actual small number of AI applications, there is only little evidence to describe the concrete implications of AI for the clinical work of physicians. Nevertheless, it can be expected that analogously to the variety of AI applications in medical disciplines, physicians’ work will change according to the task supported by the AI application. However, we see a general urgent need to integrate AI in medical education and professional training curricula [82,83].

### Fields of AI Application With the Potential to Improve Clinical Practice

Currently, AI usage in medicine is one of the most promoted topics in medicine as a new technology that will fundamentally change physicians’ clinical practice [32,84]. On the one hand, there are enormous expectations on AI-based decision support systems for better diagnosis, treatment, and clinical documentation facilitation. On the other hand, only a few AI applications have passed the regulatory requirements and have been implemented in clinical routine practice [85]. Participants in our study were optimistic that most proposed AI applications for medicine would improve patient care substantially in the future. The majority rated 23 of 25 applications positively, and 14 of these applications were evaluated by more than 70% of respondents to substantially enhance patient care. The highest potential was given for the AI application “identification of drug interactions,” while the AI application for “diagnosis of psychiatric diseases” received the least positive evaluations. We assume that due to the increasing complexity of medication administration, physicians hope to be supported by AI-based decision support systems to avoid drug interactions especially when treating polymorbid, polypharmacy patients. The low rating of AI’s potential for psychiatric diseases is remarkable as there are several AI applications for this medical field as described for ML in recent publications [52,53]. Respondents in the study population might not yet be completely informed about all potential AI applications, like in this context as well as the application of AI for speech or voice analysis using a recording.

Recently, Laguarta et al [86] from the Massachusetts Institute of Technology successfully developed and applied an AI model for diagnosing COVID-19 using only cough recordings, achieving a COVID-19 sensitivity of 98.5% with a specificity of 94.2% (area under the curve: 0.97). For asymptomatic subjects, the AI model achieved a sensitivity of 100% with a specificity of 83.2% [86]. The press release about the Massachusetts Institute of Technology Open Voice approach and the application of AI for voice analysis for the diagnosis of COVID-19 will contribute to informing physicians and the general population about less-known applications of AI for health care. To give physicians a basic understanding of AI for medical practice as well as its limitations and opportunities, Meskó and Görög [84] published a guide for medical professionals in the era of AI.

Table 2 shows that those applications using (sensor-based) continuously collected data in particular (eg, in ICUs or cardiology and neurology, imaging and video diagnostic procedures) and AI applications for workflow support were considered promising to substantially improve future patient care. In this context, Rush et al [87] argued that the data-rich ICU environment has massive AI usage potential. We can see that those fields were rated with high potential for future medicine improvement where information technology usage is high and structured data are documented. Less agreement among respondents was reached for fields with less structured data like therapeutic interviews in psychiatry.

### Personal Opinion of Physicians on the Secondary Usage of Anonymized Patient Data for AI Development and Other Research Purposes

Access to clinical research databases like the MIMIC database is an elementary precondition for AI development for medicine. We found very positive attitudes towards the secondary usage of anonymized patient data for clinical and biomedical research. The fact that many researchers are positive about the anonymization and disclosure of their data after research can be a solution to the lack of publicly open medical data. Physicians’ attitudes are in line with the general movement of science and engineering to make research data “findable, accessible, interoperable, and reusabile”—according to the FAIR data principles [88,89]. A European initiative applying the FAIR data principles is the European Open Science Cloud (EOSC), providing services to find and reuse each other’s research objects under optimal and well-defined conditions [90]. The EOSC-Hub offers a wide selection of freeware services for researchers (eg, in the fields of data management, storage, data sharing, discovery, processing, and analysis) under one federated identity security system [90].

For the question addressing the trade-off between individual data protection and improvement of medical diagnosis or therapy, no unified opinion nor tendency was found. To avoid such issues, various privacy-preserving technologies have been developed for clinical and biomedical research, such as record linkage, synthetic data generation, and genomic data privacy [91]. Price and Cohen [92] described the legal and ethical challenges of big data for data privacy and how to handle patient data for the best conception of health privacy. The authors concluded that “Privacy underprotection and overprotection each create cognizable harms to patients both today and tomorrow,” highlighting the enormous complexity of privacy in big data research.

### Strengths and Limitations

We conducted a web-based survey among hospital physicians about their opinion of AI applications for different fields of applications and the attitudes of physicians towards the secondary usage of patient data for medical research. To our knowledge, this is the first survey to interrogate physicians’ expectations and opinions of AI usage in medicine across German university hospitals. Yet, we did not investigate the usability of specific AI-based applications or decision support systems in our survey.

A limitation of our online survey is possible recruitment bias, as participating physicians may have had a particular interest or were involved in research on AI in medicine. The majority of respondents reported a positive attitude towards AI in health care. In addition, we found a positive association between self-reported technical affinity and attitude towards AI in medicine (Figure 4). Therefore, participants in our survey might have a more positive attitude towards technology and AI in health care than the whole population of physicians in German university hospitals. Even though physicians from almost every medical discipline participated in our survey, 42.2 % (128/303) were internal medicine and anesthesia department clinicians. This might have had an impact on the rating of the potential of AI application to improve health care in the future (Figure 5, Table 2). In consequence, AI applications performing tasks, which are common in these dominating disciplines, like identification of potential drug interactions and usage of continuous patient data monitoring could have received higher ratings than in a fully balanced population. Further studies on the usability and added value of AI applications in health care are needed prior to implemention in hospitals and medical practice in general. According to Magrabi et al [28], a rigorous initial and ongoing evaluation is essential for the safety and effectiveness of AI integration in sociotechnical settings like health care. Due to the huge variety and high complexity of AI applications, this paper may not take all regulatory issues into account.

### Conclusions

Most physicians expect that medicine's future will be characterized by a combination of human and artificial intelligence. The participating physicians evaluated that most of the proposed AI applications will substantially improve patient care in the future. The highest potential is given to AI applications using sensor-based, continuously collected data like electrocardiogram and electroencephalogram or continuous patient monitoring in ICUs, imaging procedures in diagnostics, and workflow support. Physicians have the greatest expectation in the use of AI for the identification of drug interactions, reflecting the rising complexity of drug administration. Thus, future clinical AI users in hospitals seem to be ready for this new technology's clinical usage. We expect that AI applications will support imaging diagnostics and that AI applications for sensor-based, continuously collected data will be used in health care in the near future. In other medical disciplines with less standardization of data processing and collection, like in the German outpatient sector, AI applications will be developed and used in clinical practice later.

In general, the secondary usage of patient data and open access to databases for medical research were seen very positively by the physicians in our survey. Researchers in clinical and biomedical research would like to benefit from better access to research databases to generate new insights for improved patient care. Thus, initiatives like the German Medical Informatics Initiative, EOSC, and FAIR data principles will improve data usage from clinical care for clinical and (bio)medical research and facilitate researchers’ access to clinical research data. In turn, that will fundamentally enhance the conditions of clinical data analysis and, as a consequence, enable better and personalized treatments for patients. Nevertheless, before new AI applications are implemented in clinical practice, the regulatory, legal, and ethical challenges must be mastered. Legislators and regulators must create the necessary framework for anonymous patient data exchange for clinical care and research, development of medical AI applications, and finally, its practical bedside use by physicians in health care.

## Appendix

Multimedia Appendix 1 Survey on the usage of artificial intelligence in stationary hospital care.

Multimedia Appendix 2 Additional tables.

Multimedia Appendix 3 Personal rating of the usage of AI in medicine? (Scale: 1 – 5; 1 = very negative; 3 = neutral; 5 = very positive).

Multimedia Appendix 4 Overall rating of artificial intelligence (AI) stratified by current occupation.

Multimedia Appendix 5 Artificial intelligence (AI) affinity score stratified by medical discipline group.

## Abbreviations

AI: artificial intelligence
DIFUTURE: Data Integration for Future Medicine
EOSC: European Open Science Cloud
ICU: intensive care unit
MIMIC: Medical Information Mart for Intensive Care
MIRACUM: Medical Informatics in Research and Care in University Medicine
ML: machine learning
SMITH: Smart Medical Information Technology for Healthcare
