# Supplementary material for: Future Medical Artificial Intelligence Application Requirements and Expectations of Physicians in German University Hospitals: Web-Based Survey
Source: J Med Internet Res. 2021 Mar 5;23(3):e26646. doi: 10.2196/26646 (PMC7980122; doi:10.2196/26646)
Supplement: Multimedia Appendix 1 [file jmir_v23i3e26646_app1.docx]

# Multimedia Appendix

**“Future Medical Artificial Intelligence Application Requirements**

**and Expectations of Physicians in German University Hospitals:**

**Web-Based Survey”**

**Survey on the usage of artificial intelligence in stationary hospital care**

The digitalisation of the health care system is a major challenge for all stakeholders involved. This concerns everyday aspects, such as the increasing use of mobile devices in clinical routine as well as developments with the potential of fundamentally transforming clinical practice, e.g. the use of artificial intelligence in medicine, like "big data analysis".

The attitude of physicians working in inpatient care towards these topics is still largely unexplored. Hence we would like to ask you as physicians to participate in the following survey. We would be grateful for your support. The survey includes 24 questions and requires 10 minutes to complete.

**Questions about artificial intelligence (AI)**

Usage of artificial intelligence (AI) in healthcare

Artificial intelligence (AI) is increasingly used in many areas of life. When AI is coupled with “big data analysis”, massive amounts of data sets can become meaningful and useful as information because the analyses of these data sets can reveal correlations we did not know before. Therefore, according to scientists the use of AI has an enormous potential to sustainably improve patient care. Thus, yet there are initial developments which use AI to make predictions about the course of a disease in certain illnesses. Also, there are already self-learning systems which evaluate images in radiology and pathology by using pattern recognition in order to support physicians in diagnostics. Plus, based on large, anonymized patient records, researchers work on the prediction of the emergence of a disease before it even causes any symptoms.
However, the effects of these developments on medical practice and in particular the (self) perception of physicians themselves have hardly been investigated, yet.

**Personal opinion about AI in healthcare**

**1) We would like to ask you for your personal opinion. Please assess to what extent you agree or disagree with the statements mentioned below. It is not a question of whether you think a statement is good or bad, but only whether you agree or disagree with the fact described.**

|  |  | Doesn’t apply at all | Rather doesn’t apply | Rather applies | Fully applies |
| --- | --- | --- | --- | --- | --- |
| **1.1** | **The usage of AI in healthcare will reduce treatment errors in the future** |  |  |  |  |
| **1.2** | **AI based decision support systems will change my work as a physician** |  |  |  |  |
| **1.3** | **The usage of AI in healthcare will increase the dependency of physicians on computerized systems** |  |  |  |  |
| **1.4** | **The benefits of AI-based systems for decision support must be proven before application in clinical practice on patients** |  |  |  |  |
| **1.5** | **The usage of AI inhibits physicians to learn a correct assessment of patients** |  |  |  |  |
| **1.6** | **The usage of AI changes the requirements to the occupational profile of physicians** |  |  |  |  |
| **1.7** | **I wish that AI will support physicians at critical decisions** |  |  |  |  |
| **1.8** | **Through the usage of AI physicians will have more time for their patients again** |  |  |  |  |
| **1.9** | **The usage of AI will affect the physician-patient relationship** |  |  |  |  |
| **1.10** | **The usage of AI will relieve me as a physician** |  |  |  |  |
| **1.11** | **The future of healthcare will be characterized by the combination of human and AI** |  |  |  |  |
| **1.12** | **AI will not be used in healthcare when the algorithms are not logically reproducible** |  |  |  |  |
| **1.13** | **Physicians should only be allowed to use AI in patient care after a dedicated professional training in this field** |  |  |  |  |
| **1.14** | **The responsibility and liability for AI-based decision recommendations must be taken over by the developers of the AI** |  |  |  |  |
| **1.15** | **AI in medicine will become a risk for patient safety** |  |  |  |  |
| **1.16** | **In the future the role of physicians will be less important for the therapy of patients** |  |  |  |  |

**4) All in all: How positive or negative do you rate the usage of AI in medicine?**

**Scale 1 – 5 (1 = very negative; 3 = neutral; 5 = very positive)**

Please select one of the following answers

 1

 2

 3

 4

 5

**Fields of application of AI in healthcare**

**2) For the following fields of application, please rate, if healthcare can be improved substantially by the usage of AI.**

|  |  | Doesn’t apply at all | Rather doesn’t apply | Rather applies | Fully applies |
| --- | --- | --- | --- | --- | --- |
| **2.1** | **Analysis of x-rays, CT, MRT, sonographies** |  |  |  |  |
| **2.2** | **Analysis of histopathologic fine-cuts** |  |  |  |  |
| **2.3** | **Analysis of endoscopic images or videos** |  |  |  |  |
| **2.4** | **Analysis of dermatologic reflected light microscopy** |  |  |  |  |
| **2.5** | **Analysis of ECGs and EEGs** |  |  |  |  |
| **2.6** | **Diagnosing rare diseases** |  |  |  |  |
| **2.7** | **Triage in emergency care** |  |  |  |  |
| **2.8** | **Diagnosing psychiatric diseases** |  |  |  |  |
| **2.9** | **Early alarm of the deterioration of patient status** |  |  |  |  |
| **2.10** | **Reduction of false alarms in intensive care medicine** |  |  |  |  |
| **2.11** | **Automatic mechanical ventilation** |  |  |  |  |
| **2.12** | **Support of enteral and parenteral nutrition** |  |  |  |  |
| **2.13** | **Oncologic therapy planning** |  |  |  |  |
| **2.14** | **Subspecification of hematologic diseases** |  |  |  |  |
| **2.15** | **Antibiotic stewardship** |  |  |  |  |
| **2.16** | **Identification of drug interactions** |  |  |  |  |
| **2.17** | **Prediction of effects of therapeutic interventions** |  |  |  |  |
| **2.18** | **Education and training of medical students and physicians** |  |  |  |  |
| **2.19** | **Assessment of prognosis of non-malignant diseases** |  |  |  |  |
| **2.20** | **Assessment of prognosis of malignant diseases** |  |  |  |  |
| **2.21** | **Medication for geriatric patients** |  |  |  |  |
| **2.22** | **Medication for pediatric patients** |  |  |  |  |
| **2.23** | **Automatic anesthesia administration** |  |  |  |  |
| **2.24** | **Workflow support in stationary hospital care** |  |  |  |  |
| **2.25** | **Medical recording, discharge letters** |  |  |  |  |

**Usage of anonymized patient data for research purposes**

Nowadays, data is considered the 21st century's oil. For the development of AI large data sets of (anonymous) patient care are required. Within the Medical Informatics Initiative of the German Federal Ministry of Education and Research (BMBF) the infrastructure to enable medical data sharing across institutional boarders is being created to eventually generate new knowledge based on this data.

**3) How do you rate the following statements concerning the usage of anonymized patient data for research purposes?**

Please rate to what extend you agree with the statements on mobile devices in hospital care below.

|  |  | Doesn’t apply at all | Rather doesn’t apply | Rather applies | Fully applies |
| --- | --- | --- | --- | --- | --- |
| **3.1** | **Anonymized patient data should be available with open access in Germany** |  |  |  |  |
| **3.2** | **The possibility to improve the treatment of diseases is more important than the individual protection of data privacy** |  |  |  |  |
| **3.3** | **I wish to be able to use a huge cross-site patient database for my own research** |  |  |  |  |
| **3.4** | **I would provide anonymized data which I have collected during my research for a collective patient database for further research purposes** |  |  |  |  |

**Biographical questions**

**Finally, we would like to ask you to answer the following biographical questions.**

**5. Your Age**

Please select one of the following answers:

 18-24 years

 25-34 years

 35-44 years

 45-54 years

 54-65 years

 >65 years

 No response

**6. Your Gender**

Please select one of the following answers:

 Female

 Male

 Diverse

 No response

**7. Your current occupation**

Please select one of the following answers:

 Assistant physician

 Medical specialist

 Senior Physician

 Clinic director

 Other________________________________________

 No response

**8. Your medical field/discipline**

Please select all applicable answers:

 Anesthesiology/intensive care medicine

 Anatomy

 Biochemistry

 Child and adolescent psychiatry and psychotherapy

 Dermatology

 Forensic medicine

 General medicine

 Gynaecology

 Human genetics

 Hygiene and environmental medicine

 Internal medicine

 Laboratory medicine

 Microbiology, virology, infectiology

 Neurology

 Neurosurgery

 Nuclear medicine

 Occupational medicine

 Ophthalmology

 Oral and maxillofacial surgery

 Otorhinolaryngology

 Paediatrics

 Pathology

 Pharmacology

 Physical and rehabilitative medicine

 Psychology

 Psychosomatic medicine

 Public healthcare

 Radiology

 Radiotherapy

 Surgery

 Transfusion medicine

 Urology

 Venereology

 Other disciplines/specialization________________________________________

**9. Your predominant workplace**

Please select all applicable answers:

 Operating Theatre

 Hospital Ward

 Outpatient Clinic

 ICU Ward

 Functional Area

 Laboratory

 Office

 Other________________________________________

**10. How many years have you been clinically active for?**

Please enter your answer here:

**________years**

**11. How pronounced would you rate your affinity to engineering and information technology?**

**Scale 1 - 5 (1 = not pronounced; 5 = very pronounced)**

Please select one of the following answers:

 1

 2

 3

 4

 5

**12. Here is space for your remarks and comments on this survey:**

Please enter your answer here:

**__________________________________________________________________________________**

**We would like to thank you very much for participating in this survey. You can now close the window of your Internet browser.**
