# Supplementary material for: Future Medical Artificial Intelligence Application Requirements and Expectations of Physicians in German University Hospitals: Web-Based Survey
Source: J Med Internet Res. 2021 Mar 5;23(3):e26646. doi: 10.2196/26646 (PMC7980122; doi:10.2196/26646)
Supplement: Multimedia Appendix 2 [file jmir_v23i3e26646_app2.docx]

# Multimedia Appendix

**“Future Medical Artificial Intelligence Application Requirements**

**and Expectations of Physicians in German University Hospitals:**

**Web-Based Survey”**

**Table S1. Categories of medical disciplines**

| **Category name** | **Medical disciplines** |
| --- | --- |
| 1. Anesthesia and intensive care medicine | - Anesthesia/intensive care medicine  - Emergency care  - Palliative care |
| 2. Surgical disciplines | - Ophthalmology  - Surgery  - Gynecology  - Otorhinolaryngology  - Oral and maxillofacial surgery  - Neurosurgery  - Urology  - Orthopedics and trauma surgery  - Dentistry |
| 3. Internal and conservative disciplines | - Internal medicine  - General medicine  - Occupational medicine  - Dermatology  - Pediatrics  - Physical and rehabilitative medicine  - Forensic medicine |
| 4. Neurological and psychiatric disciplines | - Child and adolescent psychiatry and psychotherapy  - Neurology  - Psychology  - Psychosomatic medicine |
| 5. Theoretical disciplines | - Pharmacology  - Biochemistry |
| 6. Diagnostic disciplines | - Radiology  - Laboratory medicine  - Microbiology, virology, infectiology  - Pathology  - Nuclear medicine  - Radiotherapy  - Human genetics  - Hygiene and environmental medicine |

**Table S2: Applications with the highest rated potential to improve patient care in the future (*“rather applies” and “fully applies”)**

|  | **AI Application** | **Field of application** | *n (%) |
| --- | --- | --- | --- |
| 1 | Identification of drug interactions | Medication and therapy | 280 (92.4) |
| 2 | Early alarm of the deterioration of patient status | ICU/Anaesthesia | 267 (88.1) |
| 3 | Analysis of x-rays, CT, MRT, Sonographies | Imaging procedures | 263 (86.8) |
| 4 | Analysis of ECGs and EEGs | Other diagnostic procedures | 257 (84.8) |
| 5 | Medication for geriatric patients | Medication and therapy | 243 (80.2) |
| 6 | Antibiotic stewardship | Medication and therapy | 240 (79.2) |
| 7 | Workflow support in stationary hospital care | Workflow support and education | 240 (70.2) |
| 8 | Analysis of dermatologic reflected light microscopy | Imaging procedures | 238 (78.5) |
| 9 | Medical recording or discharge letters | Workflow support and education | 237 (78.2) |
| 10 | Analysis of histopathologic fine-cuts | Imaging procedures | 235 (77.6) |
| 11 | Support of enteral and parenteral nutrition | ICU/Anaesthesia | 228 (75.2) |
| 12 | Medication for pediatric patients | Medication and therapy | 227 (74.9) |
| 13 | Automatic mechanical ventilation | ICU/Anaesthesia | 214 (70.6) |
| 14 | Education/training of medical students/physicians | Workflow support and education | 213 (70.3) |
| 15 | Diagnosing rare diseases | Other diagnostic procedures | 210 (69.3) |
| 16 | Oncologic therapy planning | Medication and therapy | 199 (65.7) |
| 17 | Reduction of false alarms in intensive care medicine | ICU/Anaesthesia | 197 (65.0) |
| 18 | Subspecification of hematologic diseases | Other diagnostic procedures | 196 (64.7) |
| 19 | Analysis of endoscopic pictures or videos | Imaging procedures | 194 (64.0) |
| 20 | Prediction of effects of therapeutic interventions | Prognosis assessment | 193 (63.7) |
| 21 | Assessment of prognosis of malignant diseases | Prognosis assessment | 192 (63.4) |
| 22 | Assessment of prognosis of non-malignant diseases | Prognosis assessment | 177 (58.4) |
| 23 | Automatic anesthesia administration | ICU/Anaesthesia | 172 (56.8) |
| 24 | Triage in emergency care | Other diagnostic procedures | 142 (46.9) |
| 25 | Diagnosing psychiatric diseases | Other diagnostic procedures | 62 (20.5) |
