# Supplementary figures and images for: Future Medical Artificial Intelligence Application Requirements and Expectations of Physicians in German University Hospitals: Web-Based Survey
Source: J Med Internet Res. 2021 Mar 5;23(3):e26646. doi: 10.2196/26646 (PMC7980122; doi:10.2196/26646)

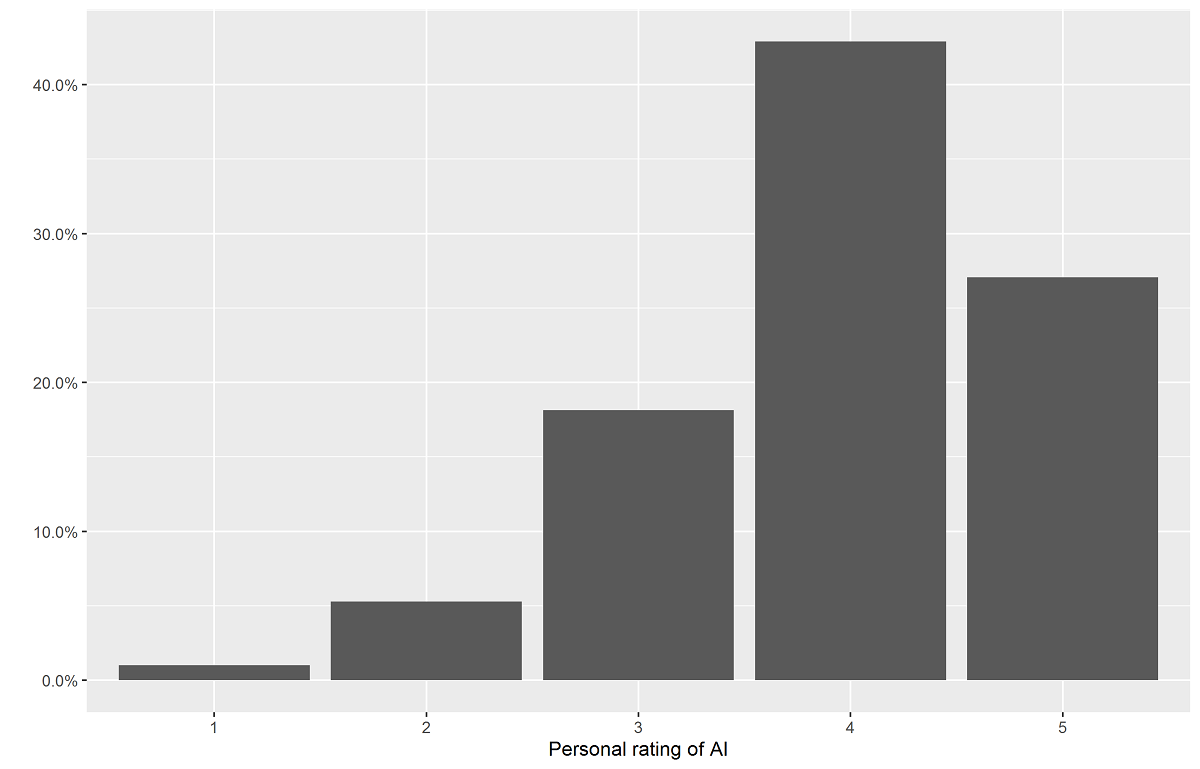

Supplement: Multimedia Appendix 3 [file jmir_v23i3e26646_app3.png]

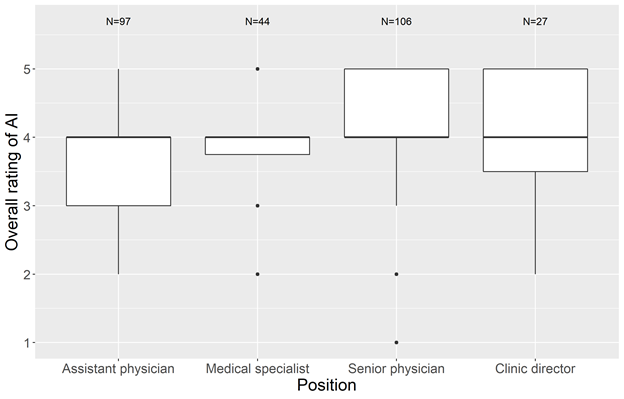

Supplement: Multimedia Appendix 4 [file jmir_v23i3e26646_app4.png]

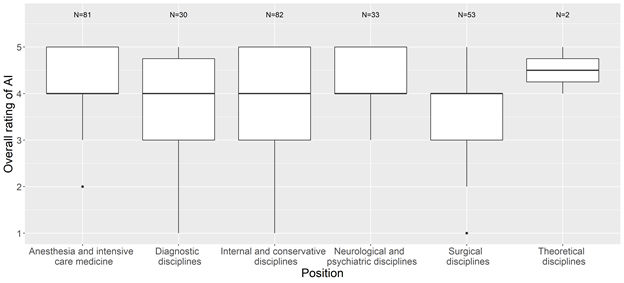

Supplement: Multimedia Appendix 5 [file jmir_v23i3e26646_app5.png]
